# Investigation of the Prevalence and Characteristics of the Retromolar Canal Using Cone-Beam Computed Tomography in a Turkish Sample

**DOI:** 10.3390/diagnostics15192526

**Published:** 2025-10-07

**Authors:** Fatoş Can, Fahrettin Kalabalık, Emre Aytuğar

**Affiliations:** 1 Department of Oral and Maxillofacial Radiology, Faculty of Dentistry, Izmir Katip Celebi University, Izmir 35640, Turkey; fatos.aykanat@ikc.edu.tr (F.C.); emre.aytugar@ikc.edu.tr (E.A.); 2 Department of Oral and Maxillofacial Radiology, Faculty of Dentistry, Sakarya University, Sakarya 54100, Turkey

**Keywords:** mandible, anatomical variation, retromolar canal, retromolar foramen, surgical anatomy, cone-beam computed tomography

## Abstract

**Background:** The aim of this study is to investigate the prevalence of the retromolar canal (RMC) and retromolar foramen (RMF) using cone-beam computed tomography (CBCT), and to evaluate the course and anatomical structure of the RMC. **Methods:** The study group consisted of CBCT images of 1008 subjects (541 females and 467 males). The prevalence and types of the RMC, as well as the frequency of the RMF, were analyzed according to age and sex. A significance level of 0.05 was accepted for all statistical analyses. **Results:** According to the findings, 575 (57.0%) RMCs and 298 (29.5%) RMFs were identified in 1008 subjects. Bilateral RMCs were observed in 327 subjects (32.4%), while unilateral RMCs were present in 248 subjects (24.6%). When 2016 retromolar regions were examined, a total of 902 RMCs and 400 RMFs were identified. No statistically significant difference was observed between the right and left retromolar regions or between sexes regarding the overall prevalence of RMCs (*p* > 0.05). The most frequently observed RMC type was Type A1, and a statistically significant difference was found between RMC types and sex. **Conclusions:** This study suggested that the RMC is a common anatomical variation that may have surgical relevance. Due to the presence of a neurovascular bundle passing through it, both the RMC and RMF should be considered in surgical and anesthetic procedures involving the retromolar region. CBCT is a reliable tool for detecting these structures and assessing their morphology.

## 1. Introduction

The retromolar canal (RMC) and its associated retromolar foramen (RMF) are considered anatomical variations in the mandibular canal, typically extending from the main mandibular canal toward the retromolar area [1,2]. These canals contain neurovascular bundles composed of myelinated nerve fibers and small-caliber vessels, contributing to sensory innervation and vascular supply of the mandibular molars, the retromolar region, and adjacent buccal mucosa [1,3]. Although often overlooked in traditional anatomy textbooks, their clinical significance is increasingly acknowledged due to the potential impact on certain oral and maxillofacial surgical procedures, including third molar extraction, sagittal split osteotomy, autologous bone graft harvesting, and dental implant placement [1,4]. Injury to these structures may lead to complications such as paresthesia, bleeding, hematoma, traumatic neuroma, and failure of local anesthesia [5].

Accurate preoperative detection of the RMC is essential for minimizing neurovascular complications. However, conventional two-dimensional imaging modalities, particularly panoramic radiography, are often inadequate for visualizing this anatomical variation. Limitations such as magnification errors, geometric distortion, and superimposition of adjacent structures hinder the detection of small anatomical variations like the RMC [6,7]. Since the diameter of the RMC can reach clinically significant dimensions in some individuals, failure to detect it on panoramic radiographs may lead to irreversible nerve damage. Therefore, in surgical interventions involving the retromolar region, the presence of this anatomical variation should ideally be evaluated, particularly in complex cases where its course may affect the procedure [6,8]. Cone-beam computed tomography (CBCT) is a widely preferred three-dimensional imaging modality for the evaluation of small and complex anatomical variations such as the RMC [9]. CBCT provides high-resolution visualization of the course and variations in the mandibular canal, offering more accurate, detailed, and superimposition-free images compared to panoramic radiographs. Moreover, due to its lower radiation dose and cost compared to conventional computed tomography (CT), it is widely used in dentistry. CBCT provides valuable information for the surgeon in various surgical procedures, such as sagittal split osteotomy, extraction of impacted third molars, and dental implant placement [10,11].

The recent literature, including systematic reviews and meta-analyses, has revealed substantial variability in RMC prevalence among different populations. Moreover, considerable discrepancies have also been reported within the same ethnic groups [1,12,13,14]. For example, Turkish CBCT-based investigations reported prevalence rates between 11% and 26.7% [5,15,16]. Such variability is considered to result from multiple factors, including ethnic and genetic diversity, environmental influences, methodological differences, and variations in sample size and study design [1]. Despite an expanding literature on the RMC, substantial gaps persist regarding its morphology, prevalence, and demographic correlates. Small samples, heterogeneous CBCT protocols, and variable canal definitions hinder the comparability and generalizability of results [12,13,17].

However, beyond these limitations, there is still a lack of studies that comprehensively investigate morphological types of the RMC according to demographic variables in a Turkish population. Previous Turkish reports mainly focused on small samples and simple prevalence rates, without providing detailed classification or age- and sex-related distributions. Therefore, the present study analyzes a large Turkish cohort and employs high-resolution CBCT to classify RMC and RMF morphology across age and sex groups. By addressing these gaps, the study aims to clarify potential anatomical and demographic variations and highlight their clinical significance for oral and maxillofacial surgery.

## 2. Materials and Methods

### 2.1. Selection of Participants

The study protocol was approved on 21 March 2024, by the Non-Interventional Clinical Research Ethics Committee of İzmir Katip Çelebi University under decision number 103. This retrospective study included CBCT images of a total of 1008 patients (541 females and 467 males) who underwent CBCT imaging for various clinical reasons between January 2015 and March 2023 at the Department of Oral and Maxillofacial Radiology, Faculty of Dentistry, İzmir Katip Çelebi University.

#### 2.1.1. Inclusion Criteria

- High-quality CBCT images.
- Subjects who had previously undergone CBCT for various clinical indications such as impacted teeth, implant surgery, and endodontic treatment.

#### 2.1.2. Exclusion Criteria

- History of surgery in the retromolar region, such as orthognathic surgery, cyst enucleation or resection.
- Presence of fractures, pathological lesions, or bone grafts in the retromolar area.
- Any artifacts that could interfere with image interpretation.
- CBCT scans in which the retromolar region was outside the field of view.
- Patients with skeletal diseases or syndromes.
- Patients with a history of head and neck surgery.
- Patients with a history of mandibular trauma.

In this study, a total of 1216 CBCT images were evaluated. After excluding 208 scans that did not meet the inclusion criteria, the remaining 1008 images were included in the analysis. The participants were divided into 4 groups according to age: ≤20 years, 21–40 years, 41–60 years, and ≥61 years.

### 2.2. Imaging Procedures

All scans were obtained in the supine position using a NewTom 5G CBCT device (Quantitative Radiology, Verona, Italy) with exposure parameters set at 110 kVp and 1–20 mA. The images were obtained using a field of view (FOV) of 12 × 8 cm and evaluated in standard resolution mode with a voxel size of 0.150 mm.

### 2.3. Image Evaluation

All CBCT images were assessed using NNT viewer software version 9.1 (QR Verona, s.r.l.; Verona, Italy) on a medical monitor Radiforce MX270W (Eizo Co.; Ishikawa, Japan) in a dark room. The presence of the RMC and RMF was evaluated on axial, sagittal, and coronal CBCT sections.

**Figure 1:**
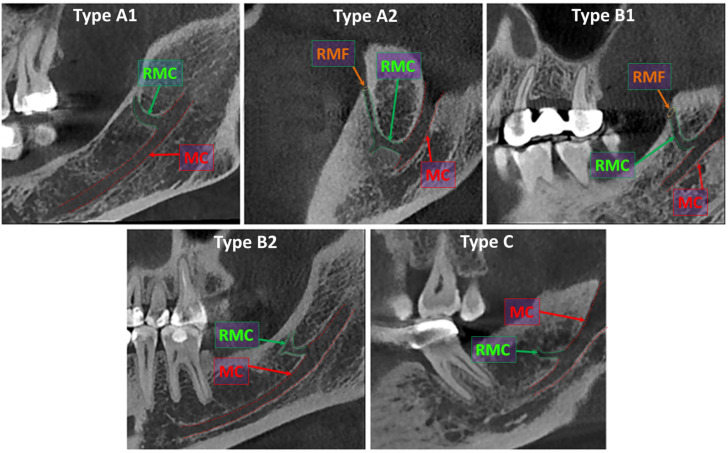
Oblique sagittal CBCT images showing the types of retromolar canals (MC: mandibular canal; RMC: retromolar canal; RMF: retromolar foramen).

The RMC was defined as an accessory canal branching off from the mandibular canal and terminating either in the retromolar region or at a foramen located on the retromolar fossa. The RMCs were analyzed according to sex and the side of occurrence. The identified RMCs were categorized according to the classification system described by von Arx et al. [7] (Figure 1). According to this classification:

1. Type A1: Retromolar canal branching vertically from the inferior alveolar canal.
2. Type A2: Retromolar canal branching vertically from the inferior alveolar canal and giving off an additional horizontal branch.
3. Type B1: Retromolar canal curving away from the inferior alveolar canal.
4. Type B2: Retromolar canal curving away from the inferior alveolar canal and giving off an additional horizontal branch.
5. Type C: Retromolar canal branching horizontally from the inferior alveolar canal.

All data were evaluated by a single oral and maxillofacial radiologist. In cases where the classification was uncertain, a second experienced radiologist was consulted, and the final decision was reached by consensus.

### 2.4. Statistical Analysis

Statistical analysis of the data obtained in the study was performed using IBM SPSS Statistics 22 (IBM Corp., Armonk, NY, USA). The distribution of the presence of the RMC and RMF, as well as the types of RMC, according to sex and age groups, was evaluated using the Chi-square test. To assess intra-examiner reproducibility, a random subset of 100 CBCT scans was re-evaluated after a 2-week interval, and agreement was quantified using Cohen’s kappa. A *p*-value of < 0.05 was considered statistically significant for all tests.

## 3. Results

In this study, the frequency of the RMC and RMF was evaluated using CBCT images of a total of 1008 individuals (mean age: 38.8 ± 17.5 years), including 541 females and 467 males, aged between 15 and 85 years. Intra-examiner reproducibility was very good (κ = 0.97). The RMC was detected in 575 individuals (57%), with unilateral presence in 248 (24.6%) and bilateral presence in 327 (32.4%). No statistically significant difference was observed in the prevalence of RMC between sexes (*p* = 0.23).

When each retromolar region was evaluated separately, a statistically significant difference was found between males and females regarding the presence of the RMC (*p* = 0.02). The prevalence of RMC in females (47.1%) was significantly higher than in males (42%) (Table 1). No significant difference was observed between the right and left sides regarding RMC prevalence (*p* = 0.21).

When the distribution of RMC types was analyzed, Type A1 (40.2%) and Type B1 (38.9%) were the most common, while Types A2, B2, and C were less frequent (0.4%, 8.9%, and 11.5%, respectively). Sex-related differences were observed: Type A1 was more frequent in females, whereas Type B1 predominated in males (*p* = 0.018) (Table 2).

RMFs were identified in 298 patients (29.6%). Among them, 40.9% were located on the right side, 24.8% on the left side, and 34.2% bilaterally. No sex-related difference was observed (*p* = 0.586) (Table 3). RMF was present in 44.3% of the retromolar regions where an RMC was detected.

Analysis of age groups showed a significantly higher prevalence of RMC in the 41–60 and ≥61 age groups compared to ≤20 and 21–40 years (*p* = 0.000). The lowest prevalence was observed in individuals ≤20 years (Table 4). Regarding canal type distribution, B1 predominated in ≤20 years; both A1 and B1 were common in 21–40 years; B1 predominated in 41–60 years; and A1 was most frequent in ≥61 years. Notably, A2 was found only in the 41–60 group (Table 5).

## 4. Discussion

Anatomical variations in the inferior alveolar nerve have long garnered attention in the literature. The RMC represents a bifid variation in the inferior alveolar nerve, branching from the mandibular canal in the retromolar region and usually emerging into the retromolar fossa through the RMF [7,18,19]. Neurovascular structures passing through the RMC supply the mandibular molar teeth, buccal mucosa, posterior part of the alveolar process of the mandible, mucosa over the retromolar fossa, and occasionally the temporalis and buccinator muscles [13]. The neurovascular contents of the RMC should be considered during surgical procedures involving the posterior mandible. Failure to identify the RMC in the preoperative period may increase the risk of complications during intraoral procedures [5]. However, this risk largely depends on factors such as the canal’s morphology, its position, and the type of surgical procedure. The buccal positioning and curved morphology of the RMC may complicate certain surgical procedures and increase the risk of complications depending on the surgical approach [14]. Hemorrhage from its vascular structures may restrict visibility within the surgical field, while potential injury to associated neural components, particularly during procedures such as ramus osteotomies, autologous bone harvesting, and extraction of impacted third molars, may compromise surgical outcomes in some cases [7,20,21]. Reported complications include paresthesia, traumatic neuroma, bleeding, hematoma, and ecchymosis [5].

Studies investigating the prevalence of the RMC in cadavers, panoramic radiographs, and CBCT images reported considerable variation in detection rates. This variability is most likely attributable to methodological and technical differences among the studies. Research based on panoramic radiographs reported relatively low detection rates, ranging from 0% to 16.9% [8,20]. In contrast, wet cadaver studies have demonstrated significantly higher prevalence, ranging from 40% to 72%, as dissection and histological examination allow direct identification of fine anatomical details [22,23]. The primary reason for the lower detection rates in panoramic radiographs is the inherent limitations of the imaging technique, including its two-dimensional nature, anatomical superimposition, and geometric distortion. These factors significantly hinder the visualization of small and intricate anatomical structures such as the RMC [24,25].

International studies using CBCT and CT have reported prevalence rates of the RMC ranging from 7.7% to 75.4% across different populations. Interestingly, even studies conducted within the same ethnic population have reported markedly different prevalence rates. For instance, RMC prevalence in Iran ranged from 14.5 to 22.0% [26,27], while in Egypt, it was 11.2% [17]. Indian cohorts demonstrated a wide range at 23.1–72.5% [14,28]. In Brazil, prevalence ranged from 7.7 to 24.5% [29,30], and in Spain it was 23.1% [31]. Lower frequencies were reported in Korea at 8.5% [32]. Japanese studies reported the widest range at 25.6–75.4% [7,20,33]; in China it was 25.9% [34], and in Poland 10.5% [2]. Studies conducted in Turkey reported rates ranging from 11.0 to 26.7% [3,6,16]. The present study identified a considerably higher prevalence of 57.0% in 1008 CBCT scans. This discrepancy is most likely multifactorial. First, the use of high-resolution CBCT with a small voxel size in the present study may have allowed the detection of finer accessory canals that could be overlooked with larger voxel sizes. Second, the inclusion of all morphological variants according to a detailed classification system and the lower diagnostic thresholds applied in this study probably improved detection sensitivity compared to previous reports. Third, population-specific anatomical characteristics may have contributed to the higher rate observed in our cohort. Observer experience and careful image evaluation may have further enhanced identification accuracy. These combined factors help explain the substantially higher prevalence observed in our study compared with most previous reports.

The literature indicates no significant difference in RMC prevalence between sexes [7,30,31,32,34]. In our study, the prevalence of the RMC was 58.8% in females and 55% in males. Consistent with the literature, no statistically significant difference was observed between sexes regarding RMC prevalence.

The classification and nomenclature of the RMC remain controversial. While some researchers consider the RMC to be a variant of the mandibular canal that terminates in the retromolar region [35,36], others argue that it represents a distinct anatomical structure with clinical significance [7,32,37]. Although various classifications of RMC types have been proposed in the literature, the system introduced by von Arx et al. [7] has been widely adopted by many researchers. Using this classification, B1 or A1 typically predominates. Komarnitki et al. [38] identified B1 as most common. Shogo Kikuta et al. [20] reported B1 at 66.7% and A1 at 26.7%, with no A2 or B2. In contrast, Filo et al. [15] found A1 at 39.82% and B1 at 24.07%, while von Arx et al. [7] reported A1 at 41.9%, B1 at 29%, A2 at 16.1%, and B2 at 12.9%, with no type C. These findings suggest that the von Arx classification demonstrates similar distribution patterns across different populations. However, these studies did not provide detailed or statistical analyses of sex-based differences in RMC type distribution. In the present study, the observed prevalence rates were as follows: type A1 (40.2%), type B1 (38.9%), type C (11.5%), type B2 (8.9%), and type A2 (0.4%). Additionally, sex-based analysis revealed that type A1 was significantly more common in females, whereas type B1 was significantly more prevalent in males. In this regard, our study makes a valuable contribution to the existing literature employing the von Arx classification by providing novel insights into sex-specific variations in RMC types.

The distribution of the RMC across different age groups has been addressed in only a limited number of studies in the literature. In their study, Miguel Puche-Roses et al. [31] divided individuals into four age groups and reported the highest RMC prevalence in the 18–29 age group (38.5%). They observed a gradual decline in prevalence with increasing age; however, the differences among age groups were not significant. Similarly, von Arx et al. [7] found the highest frequency of RMC in the 21–40 age group, yet no statistically significant association with age was reported. In contrast, our study identified a significant relationship between RMC prevalence and age group. Specifically, the prevalence of RMC was significantly higher in the 40–60 and ≥60 age groups compared to younger individuals, whereas it was significantly lower in individuals aged ≤20 years. These findings suggest that the detectability of RMC increases with age. Furthermore, to our knowledge, no previous studies have investigated the distribution of RMC types according to age groups. In this regard, our study provides additional value to the literature. According to the findings, type B1 was the most frequently observed RMC variant among individuals aged ≤20 years. In the 21–40 age group, types A1 and B1 were detected at similar frequencies. In the 41–60 age group, type B1 remained the most prevalent subtype, whereas in individuals aged ≥61, type A1 was the most frequently identified. Notably, type A2 was observed only in the 41–60 age group.

The RMF is a rare anatomical variation located on the alveolar surface of the molar triangle in the posterior mandible. It typically represents the external opening of the RMC, which branches off from the mandibular canal [24]. Failure to recognize the RMF prior to flap elevation may result in neurovascular injury and paresthesia. As with the RMC, the RMF represents a variation with potential implications for oral surgical procedures [39]. Studies investigating the prevalence of the RMF, similar to those on the RMC, have reported highly variable results. The reported prevalence of RMF ranges from 3.2% to 72% in dry mandible studies, from 5.4% to 75.4% in CBCT studies, and from 3.06% to 8.8% in panoramic radiographic studies [5]. This wide range has been attributed to factors such as ethnic differences, environmental and genetic influences, methodological variations, and discrepancies in sample sizes among studies [40].

Previous studies have reported no statistically significant difference in the prevalence of RMF between males and females [7,39,41,42]. Consistent with the literature, our study also found no significant difference in the distribution of RMF according to sex.

One of the major strengths of this study is the evaluation of a large sample group using high-resolution CBCT images. The detailed analysis of RMC types by sex and age groups provides statistically supported data on a subject that has rarely been addressed in the literature. Determining the prevalence and morphological diversity of the RMC and RMF is of substantial clinical importance, particularly for surgical procedures involving this region, thereby enhancing the practical value of the study. However, this study has certain limitations. First, as the primary aim of the study was to determine prevalence and morphological classification of the RMCs, quantitative measurements of canal diameter and length were not performed, and the analysis was restricted to identifying and classifying them. Second, the contents of the RMC were not histologically verified, and the evaluation was based solely on radiological imaging. Finally, since this study was based on CBCT scans obtained for specific clinical indications (such as implant planning, impacted third molars, or suspected pathologies), a potential selection bias may exist. Therefore, the findings may not be fully generalizable to the general population. Nevertheless, the large sample size and the inclusion of patients across a wide age range enhance the reliability and representativeness of the results.

## 5. Conclusions

The present study demonstrates that the RMC and RMF are common anatomical variations with diverse morphologies. Their presence should be considered during preoperative planning, as undetected canals may increase the risk of neurovascular injury and failure of local anesthesia. CBCT enables reliable identification and course delineation of these structures. Based on these findings, routine preoperative CBCT assessment of the retromolar region should be considered before mandibular third-molar surgery, sagittal split osteotomy, autologous ramus graft harvesting, and posterior-mandibular implant placement to reduce the risk of neurovascular injury and anesthesia failure. Nevertheless, the findings should be interpreted with caution due to certain limitations, including the absence of histological verification, lack of quantitative measurements of canal diameter and trajectory, and the single-center design based on a Turkish population. Future multicenter studies incorporating anatomical and histological validation, as well as quantitative analysis, are needed to confirm these findings and enhance their clinical relevance.

## Figures and Tables

**Table 1:** Distribution of the prevalence of retromolar canals (RMC) by sex in retromolar regions.

RMC	Sex				*p*
	Female		Male		
	*n*	(%)	*n*	(%)	
Present	510	(47.1)	392	(42.0)	0.02
Absent	572	(52.9)	542	(58.0)	

Chi-square test.

**Table 2:** Distribution of RMC types by sex.

RMC Types	Sex				*p*
	Female		Male		
	*n*	(%)	*n*	(%)	
A1	223	(43.7) ^a^	140	(35.7) ^b^	0.018
A2	4	(0.8) ^a^	0	(0.0) ^a^	
B1	180	(35.3) ^a^	171	(43.6) ^b^	
B2	41	(8.0) ^a^	39	(9.9) ^a^	
C	62	(12.2) ^a^	42	(10.7) ^a^	
Total	510	(100)	392	(100)	

Chi-square test with Bonferroni-adjusted post hoc comparisons. In each row, values with the same superscript letter do not differ significantly; different letters indicate significant differences at α = 0.05.

**Table 3:** Distribution of RMF prevalence according to sex.

RMF	Sex				*p*
	Female		Male		
	*n*	(%)	*n*	(%)	
Present	156	(28.8)	142	(30.4)	0.586
Absent	385	(71.2)	325	(69.6)	
Total	541	(100)	467	(100)	

Chi-square test.

**Table 4:** Prevalence of RMC by age group.

RMC	Age Groups								*p*
	≤20 Years		21–40 Years		41–60 Years		≥61 Years		
	*n*	(%)	*n*	(%)	*n*	(%)	*n*	(%)	
Present	67	(20.6) ^a^	319	(42.1) ^b^	368	(55.3) ^c^	148	(55.6) ^c^	0.000
Absent	259	(79.4) ^a^	439	(57.9) ^b^	298	(44.7) ^c^	118	(44.4) ^c^	
Total	326	(100)	758	(100)	666	(100)	266	(100)	

Chi-square test with Bonferroni-adjusted post hoc comparisons. Within rows, identical superscript letters indicate no significant differences among age groups; different letters indicate *p* < 0.05.

**Table 5:** Prevalence of RMC types by age group.

Age Groups	RMC Types	*n*	(%)
≤20 years	A1	24	(7.4)
	B1	31	(9.5)
	B2	2	(0.6)
	C	10	(3.1)
	Total	67	(20.6)
21–40 years	A1	132	(17.4)
	B1	132	(17.4)
	B2	32	(4.2)
	C	23	(3.0)
	Total	319	(42.1)
41–60 years	A1	135	(20.3)
	A2	4	(0.6)
	B1	146	(21.9)
	B2	40	(6.0)
	C	43	(6.5)
	Total	368	(55.3)
≥61 years	A1	72	(27.1)
	B1	42	(15.8)
	B2	6	(2.3)
	C	28	(10.5)
	Total	148	(55.6)

## Data Availability

The data presented in this study are available on request from the corresponding author due to privacy.
